# Diet and food insecurity among mothers, infants, and young children in Peru before and during COVID‐19: A panel survey

**DOI:** 10.1111/mcn.13343

**Published:** 2022-03-11

**Authors:** Rebecca Pradeilles, Rossina Pareja, Hilary M. Creed‐Kanashiro, Paula L. Griffiths, Michelle Holdsworth, Nervo Verdezoto, Sabrina Eymard‐Duvernay, Edwige Landais, Megan Stanley, Emily K. Rousham

**Affiliations:** ^1^ Centre for Global Health and Human Development, School of Sport, Exercise and Health Sciences Loughborough University Loughborough UK; ^2^ Instituto de Investigación Nutricional Lima Perú; ^3^ School of Clinical Medicine University of the Witwatersrand Johannesburg South Africa; ^4^ Montpellier Interdisciplinary Centre on Sustainable Agri‐Food Systems Univ Montpellier, CIRAD, CIHEAM‐IAMM, INRAE, Institut Agro, IRD (UMR MoISA) Montpellier France; ^5^ School of Computer Science and Informatics Cardiff University Cardiff UK

**Keywords:** breast feeding, COVID‐19, diet, food insecurity, infant, pandemic, women's health

## Abstract

The COVID‐19 pandemic may impact diet and nutrition through increased household food insecurity, lack of access to health services, and poorer quality diets. The primary aim of this study is to assess the impact of the pandemic on dietary outcomes of mothers and their infants and young children (IYC) in low‐income urban areas of Peru. We conducted a panel study, with one survey prepandemic (*n* = 244) and one survey 9 months after the onset of COVID‐19 (*n* = 254). We assessed breastfeeding and complementary feeding indicators and maternal dietary diversity in both surveys. During COVID‐19, we assessed household food insecurity experience and economic impacts of the pandemic on livelihoods; receipt of financial or food assistance, and uptake of health services. Almost all respondents (98.0%) reported adverse economic impacts due to the pandemic and 46.9% of households were at risk of moderate or severe household food insecurity. The proportion of households receiving government food assistance nearly doubled between the two surveys (36.5%–59.5%). Dietary indicators, however, did not worsen in mothers or IYC. Positive changes included an increase in exclusive breastfeeding <6 months (24.2%–39.0%, *p* < 0.008) and a decrease in sweet food consumption by IYC (33.1%–18.1%, *p* = 0.001) and mothers (34.0%–14.6%, *p* < 0.001). The prevalence of sugar‐sweetened beverage consumption remained high in both mothers (97%) and IYC (78%). In sum, we found dietary indicators had not significantly worsened 9 months into the COVID‐19 pandemic. However, several indicators remain suboptimal and should be targeted in future interventions.

## Key messages

- Almost all respondents (98.0%) reported negative economic impacts on their households from the pandemic.
- During the pandemic, almost half of households (46.9%) were at risk of moderate or severe household food insecurity.
- During the pandemic, 66.9% of households received cash transfers, and 59.4% received food assistance from the government.
- Nine months into the pandemic, positive changes included an increased prevalence of exclusive breastfeeding <6 months, a decreased proportion of mothers and infants and young children (IYC) consuming sweet foods, and stable dietary indicators for IYC and women.
- Consumption of sugar‐sweetened beverages and unhealthy foods remained high across the two time periods for mothers and IYC and should be targeted in future interventions.

## Graphical abstract

We aimed to assess whether the COVID‐19 pandemic influenced diets of mothers and infants and young children (IYC) in two low‐income urban areas of Peru. We found that, compared to prepandemic data, maternal and IYC dietary indicators had not significantly worsened nine months into the pandemic. A high proportion of households received food assistance or government cash transfers during the pandemic which may have contributed to the stability of dietary indicators. However, indicators such as sweet food consumption (despite reducing during the pandemic) and sugar‐sweetened beverages remained high across the two time periods and should be targeted in future interventions.

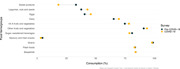

## 1 INTRODUCTION

Despite significant progress over the last decades, many low‐ and middle‐income countries (LMICs) still encounter high levels of malnutrition, often including multiple burdens of underweight, stunting, micronutrient deficiencies, and overweight/obesity. The potential risks of the COVID‐19 pandemic on maternal and infant nutrition have been highlighted, with all forms of malnutrition predicted to increase due to the pandemic (Headey et al., 2020).

The COVID‐19 pandemic is expected to adversely affect infant and young child (IYC) nutrition in LMICs through a combination of increased household food insecurity, lack of access to nutrition‐related health services, and poorer quality diets (Picchioni et al., 2021). Diet quality may be worsened by lower dietary diversity; changes in the types and quantities of foods consumed, or changes in the number of meals consumed. Reduced diet quality may in turn be driven by unemployment, worsening economic conditions, and reduced access to food (Akseer et al., 2020; Pérez‐Escamilla et al., 2020; Picchioni et al., 2021). In such crises, IYC are at increased nutritional risk because of the high energy and nutrient requirements needed to support healthy growth and development and the potential for long‐term adverse health consequences (Lutter et al., 2021).

A recent systematic review of the effects of the COVID‐19 pandemic on diet quality, food security (FS), and nutritional status found major gaps in evidence on outcomes such as dietary diversity of women and young children (Picchioni et al., 2021). Furthermore, evidence indicates that the pandemic has increased the proportion of households with moderate or severe food insecurity in LMICs, mainly through loss of income. Household food insecurity can potentially lead to increases in different forms of malnutrition (Rocha et al., 2021), such as iron or other micronutrient deficiencies, as well as overweight and obesity or other nutrition‐related noncommunicable diseases, which in turn increases the risk of COVID‐19 morbidity and mortality (Gao et al., 2021).

The COVID‐19 outbreak has also highlighted stark inadequacies in pandemic preparedness. Weaknesses in food and health systems across the globe have led to a lack of protection against hunger, food, nutrition, and health insecurity in the most vulnerable groups (Pérez‐Escamilla et al., 2020) and reduced access to health services (Headey et al., 2020). Policy responses (e.g., financial support/social protection) have mitigated some of the adverse effects of the pandemic on malnutrition, but data on the efficiency of policy responses on nutritional outcomes are scarce (Picchioni et al., 2021).

Concerns have been raised regarding the adverse impacts of the pandemic on overall breastfeeding prevalence due to isolation of mothers, lack of breastfeeding counselling due to disrupted health services, and less access to enabling environments outcomes (Busch‐Hallen et al., 2020; Picchioni et al., 2021). However, many aspects surrounding the impact of the pandemic on the diets of mothers and IYC remain poorly understood (Picchioni et al., 2021).

Peru has made significant progress in reducing undernutrition in the last 20 years, with the prevalence of stunting among under‐fives reduced from 30.0% to 13.1% between 2007 and 2016 (Huicho et al., 2016, 2020), and a further decline to 12.1% in 2019 (INEI, 2020a). With increasing economic development, however, overweight and obesity prevalence rates in adults have increased rapidly. Nationally, 62.9% of women of reproductive age (WRA) are overweight (39.5%) or obese (23.4%) (INEI, 2021), compared to 34% overweight and 14.1% obese in 2008 (Poterico et al., 2012). The prevalence of anaemia in WRA and IYC also remains high (INEI, 2020a) with 40.0% of children aged 6–36 months experiencing anaemia in both 2019 and 2020 (INEI, 2020a, 2021). Peru has a rapidly expanding urban population, comprising 79.3% of the total population in 2017 (INEI, 2017). Multiple forms of malnutrition (stunting, anaemia, and overweight/obesity), coexist and are a major health concern in urban centres, particularly among low socioeconomic sectors.

Peru went into a rapid lockdown and declared a state of emergency in response to the COVID‐19 pandemic in March 2020. Despite rapid action, the country has experienced the highest mortality rates from COVID‐19 globally (Dyer, 2021). Subsequently, the country went through varying periods of lockdown and easement, resulting in a drastic reduction in the availability of health services as well as exposing long‐term healthcare inequalities (Gianella et al., 2021). IYC in Peru are offered routine “well child” (*Crecimiento y Desarrollo* [CRED]) health services which include growth and development monitoring, health and nutrition counselling, vaccination, haemoglobin measurement, and iron supplementation. In the initial months of the state of emergency, health services ceased to offer face‐to‐face CRED appointments, with some gradual resumption of face‐to‐face services from August 2020 alongside virtual/telephone consultations.

Studies on the impact of COVID‐19 on diet and nutrition are often limited by the lack of directly comparable data immediately before the pandemic. Further, surveys during the pandemic have often relied on the voluntary completion of online questionnaires or telephone interviews, yielding results that are susceptible to self‐selection bias and underrepresentation of disadvantaged communities who are most affected by pandemic economic shocks. The primary aim of this study is to assess whether the COVID‐19 pandemic influenced dietary indicators of mothers and IYC living in two low‐income urban areas of Peru by direct comparison pre‐ and during the pandemic via a panel survey. In the survey during COVID‐19, we assessed household food insecurity experience; the perceived economic impacts of the pandemic on households’ livelihoods, and receipt of financial or food assistance and uptake of health services to understand the broader contextual influences on mothers and IYC diets in these disadvantaged communities.

## 2 METHODS

### 2.1 Study design and sampling

This was an unbalanced panel study conducted amongst low‐income urban households in Peru. We collected data via two surveys: one conducted from December 2019 to March 2020 before the COVID‐19 pandemic (PERUSANO survey) and one conducted in December 2020 (STAMINA survey), 9 months after the onset of the pandemic. The PERUSANO survey was part of a wider interdisciplinary project to address multiple forms of malnutrition, particularly stunting, anaemia, and risk of overweight/obesity, in urban Peru while the STAMINA survey was designed to examine the nutritional risks of mothers and IYC in the same communities during COVID‐19. The surveys took place in Manchay, Pachacamac District, Lima, and the city of Huánuco, Huánuco District in the Andean Highlands. In each study area, we purposively selected the principal health centre and one subsidiary health centre. Periurban (low‐income urban) communities within the jurisdiction of these health centres were selected to participate.

In the PERUSANO survey, we recruited participants by systematic random sampling using quotas via house‐to‐house visits. Before recruitment, enumerators mapped each block of houses within each “sector” (local planning administrative unit for urban areas) of the community within the health centre catchment, and the block was used as the sampling unit. We chose a block at random as the starting point from the mapped sector, then proceeded to knock on the first house, and every third house thereafter until completing the sector. A random starting point was chosen for the next sector, and recruitment continued. The target sample size for PERUSANO was 360 mother–infant dyads based on the original aims before the emergence of COVID‐19. The sample size rationale was to capture the diversity of characteristics relevant to IYC diets, food patterns, and practices via quota sampling by age (6–11, 12–17, and 18–23 months), maternal employment (employed/nonemployed) and study area (Lima/Huánuco). Recruitment stopped in March 2020 because of the pandemic, at which point 244 mother–infant dyads had been recruited.

For the STAMINA survey, the sample size was set to match the pre‐COVID sample (*n* = 250) with the same quota sampling by age and study site. Sampling took place through follow‐up of the pre‐COVID‐19 survey participants (PERUSANO) for those still eligible (aged 6–23 months) (24% of the sample). We recruited the remaining participants using systematic random sampling from the local authority child registration records for the participating health centres. From the total number of eligible IYC records within an age group, we selected every *n*th record to attain the required number of participants. At least two attempts were made to contact participants via telephone before noting them as unavailable. If one participant declined or was unavailable, the next birth record after that case was selected.

In both surveys, a screening questionnaire was used to check the eligibility of mothers and IYC with the following inclusion criteria: i. singleton infants aged ≥6 months and <24 months on the day of interview; ii. no congenital malformations affecting nutrition or growth; and iii. primary residence of mother/primary caregiver in the study site for the previous 6 months.

### 2.2 Data collection

For both surveys, data collection took place using structured questionnaires with precoded responses, administered by trained enumerators with >10 years’ experience in conducting community‐based health, demographic, and nutrition surveys. Interviews were conducted face‐to‐face via household visits (pre‐COVID‐19) and by telephone (during COVID‐19). The data collection team underwent a 2 weeks’ training programme before each of the two surveys. For both surveys, the questionnaire was produced in English and translated to Spanish, and rechecked in both languages by a team member fluent in both languages. For PERUSANO, questionnaires were piloted in Lima (*n* = 20 interviews) using mothers of IYC living in another similar community (Canto Grande) to the target communities and changes were made accordingly. In the pre‐COVID survey, two enumerators worked as a pair on household visits in Manchay, Lima, and a further two in Huánuco. Two experienced supervisors, one in Manchay and one in Huánuco, checked the completed questionnaires at the end of each week for quality control and to ensure there were no missing data. For STAMINA, new questions were piloted via telephone with 15 caregivers. The telephone interviews during COVID‐19 were conducted by the same team of four enumerators with one additional enumerator. One supervisor from the original pre‐COVID‐19 team checked the completed questionnaires. Hence, the same team was used for both surveys and had close knowledge of the communities from which participants were selected.

For the PERUSANO survey, paper questionnaires were completed, and data were entered in Microsoft Access. Automated consistency checks were run and double data entry for a random sample of 10% of questionnaires was used to calculate the error rate. A predefined threshold was set to determine an acceptable level of variation in responses, with the error rate set at 1%. For the STAMINA survey, questionnaires were completed using tablets (Samsung Galaxy Tab‐A) with an electronic data capture (CsPRO) during telephone interviews. Data quality was enhanced in CSPrO through programming of skips, controls, and other logic of the questionnaire.

Data collected in both surveys included: household sociodemographic characteristics; infant and young child feeding (IYCF) practices using standardised and validated questions (WHO, 2008; WHO and UNICEF, 2021), and child and maternal qualitative 24‐h dietary recalls using the standardised open recall method (WHO, 2008). These questionnaire modules were the same in both surveys. New questions were added in the STAMINA questionnaire to assess the impact of COVID‐19 on households including changes in employment status, adaptations to finance, sources of financial support, household food insecurity experience as well as access to, and uptake of, well‐child clinics and vaccination health services. We developed questions through engagement with policy‐makers, stakeholders, and expert discussion forums that highlighted gaps in knowledge and concerns surrounding the potential impacts of the pandemic in Peru. We also reviewed emerging literature on the consequences of COVID‐19 on maternal and infant nutrition in LMICs. We measured the experience of household food insecurity using the validated food insecurity experience scale (FIES) survey module (Cafiero et al., 2018).

### 2.3 Data management and analyses

#### 2.3.1 Diet outcomes

For both surveys, we used the most recent guide to generate IYCF practices for infants aged 0–23 months (WHO and UNICEF, 2021). Breastfeeding indicators (i.e., ever breastfed; early initiation of breastfeeding; exclusive breastfeeding under 6 months; continued breastfeeding 12–23 months) were generated using the retrospective recall of the mother/caregiver. Given that our sample of IYC only included those aged 6–23 months, we were not able to generate the exclusive breastfeeding indicator using 24‐h recall as advised in the IYCF guide. Instead, we asked a series of questions to establish retrospectively whether mothers practised exclusive breastfeeding in the first 6 months or whether foods and beverages were introduced early. Complementary feeding indicators (i.e., introduction of solid, semisolid or soft foods; dietary diversity score; minimum dietary diversity [MDD]; minimum meal frequency [MMF]; minimum acceptable diet [MAD]; egg and/or flesh food consumption; sweet beverage consumption; unhealthy food consumption; and zero vegetable or fruit consumption) were generated using mother/caregiver reported intakes of foods and beverages during the past 24 h.

For the mother/caregiver, the reported food and beverage consumption during the past 24 h was used to calculate dietary diversity scores (FAO and FHI 360, 2016). Women who had consumed at least 5 out of the 10 predefined food groups were classified as meeting the adequate MDD for women. We also derived standardised maternal indicators of: egg and/or flesh food consumption, zero fruit and vegetable consumption, sweet beverage consumption, and unhealthy food consumption to match with the IYC indicators.

Full definitions of maternal and IYC dietary indicators are provided in Supporting Information Appendix 1.

#### 2.3.2 Household food insecurity

The FIES is an eight‐item experience‐based scale of food insecurity severity. Respondents answered (yes/no) questions on household food‐related behaviours and experiences of limited access to food due to lack of resources in the past 30 days. Statistical techniques borrowed from the toolkit of item response theory (Rasch models) allowed the generation of two prevalence rates comparable across countries: i. moderate or severe food insecurity and ii. severe food insecurity only (Cafiero et al., 2018). The internal reliability of the instrument was good as the modified Rasch reliability test was estimated at 0.80 (Agarwal et al., 2009).

#### 2.3.3 Sociodemographic factors

We created a common household wealth index for both surveys using factor analysis (i.e., multiple correspondence analysis) applied to proxy indicators of the household environment (ownership of consumer durables; source of drinking water and type of toilet facilities; the number of household members per room used for sleeping; type of materials used for the floors, roof, and walls; and livestock ownership). Analysis of proxy indicators of the household environment revealed no major differences between Lima and Huánuco; hence, we ran the factor analysis with the two settings combined. Variables with low variability (i.e., either less than 5% or more than 95% ownership) were not included in the factor analysis. We split the continuous score of the household wealth index into tertiles of socioeconomic status (SES), with the first tertile representing the relatively poorest households. The first component retained explained 85% of the overall variance. We assessed internal validity by tabulating ownership of durable assets and other housing characteristics by SES tertile. We categorised mothers’ self‐report of completed educational level as less than secondary; secondary/technical, and university level. Other sociodemographic characteristics included maternal working status (yes/no); marital status (married/living together vs. not); child's age and sex; maternal age; and place of residence (Lima vs. Huánuco).

#### 2.3.4 COVID‐19 pandemic related factors

From the survey during COVID‐19, we collated responses to questions on financial impacts of the pandemic on households, adaptations to financial impacts, sources of financial support, as well as access to and uptake of nutrition‐related health services reported by the caregiver. Questions regarding sources of food assistance were asked in both surveys.

### 2.4 Statistical analyses

We generated descriptive statistics (mean, standard deviation or number [*n*], and percent) for sociodemographic and maternal and infant nutrition‐related factors for each survey. For the STAMINA survey, we described the additional variables on the impact of the pandemic on households and livelihoods, that is, economic impact; coping mechanisms; household food insecurity; and access and uptake of nutrition‐related health services. To examine whether the pandemic influenced dietary outcomes, we performed univariate and multivariable logistic regressions for binary outcomes and linear regressions for continuous outcomes, with the survey type pre‐COVID‐19 (PERUSANO) versus during COVID‐19 (STAMINA) as the main exposure variable. To account for repeated measures (24% of the sample) across the two surveys, we used a mixed‐effects model with a random effect (intercept). Models were adjusted for competing exposures (i.e., those not related to the exposure but the outcomes only) as adjusting for these increases precision in estimates of exposure–outcome associations. These competing exposures included maternal education; maternal working status; wealth index; and place of residence. Models for maternal and child outcomes controlled for maternal age or child age, respectively. We used Stata SE version 16 for statistical analyses. The type I error risk was set at 0.05.

### 2.5 Ethics Statement

Ethical approval for the PERUSANO project was obtained from the Ethical Review Committee of the Instituto de Investigación Nutricional (IIN), Peru (Reference 388‐2019/CIEI‐IIN) and Loughborough University (C19‐87). Written informed consent was provided by all participants after receiving written and verbal information about the study. For the STAMINA project, ethical approval was obtained from IIN (Reference 270‐2020/PROY.367) and Loughborough University (Reference 1926). Verbal consent was provided by mothers or other caregivers following guidelines for research under COVID‐19 of the Ethical Research Committee of IIN. The project was also registered with the National Institute of Health under the requirement for all studies related to COVID‐19 (PRISA Reference EI00000001577). In both surveys, participants were informed of the right to withdraw from participation.

## 3 RESULTS

### 3.1 Sociodemographic characteristics of the study samples

The overall sample of mother–infant dyads that met the inclusion criteria across the two surveys was 498 (*n* = 244 for PERUSANO and *n* = 254 for STAMINA). Response rates for inclusion into the study of eligible participants were 61% and 76% for PERUSANO and STAMINA, respectively. Overall, 85.7% of women were married or cohabiting and 57.9% had a secondary level of education. There was an equal distribution of participants between Lima and Huánuco (Table 1). The mean age of mothers and their offspring was 30.0 years (6.5) and 15.0 months (5.3), respectively. Most sociodemographic characteristics were balanced between the two samples apart from maternal working status. Pre‐COVID‐19, 32.8% of women reported working versus 21.7% during the pandemic.

**Table 1:** Sociodemographic characteristics of the sample

	PERUSANO (pre‐COVID‐19) (*n* = 244)	STAMINA (COVID‐19) (*n* = 254)	*p* a
*Continuous variables*	*Mean* (SD)	*Mean* (SD)	
Maternal age (years)	30.0 (7.0)	30.0 (6.1)	0.66
Child's age (months)	15.0 (5.3)	15.0 (5.2)	0.94

*Categorical variables*	*n (%)*	*n (%)*	
Child's sex (% males)	129 (52.9)	129 (50.8)	0.64
Place of residence			
Lima	125 (51.2)	128 (50.4)	0.85
Huánuco	119 (48.8)	126 (49.6)	
Maternal working status (yes)	80 (32.8)	55 (21.7)	0.005
Marital status (married/living together)	212 (86.9)	214 (84.6)	0.46
Maternal education			
Less than secondary	80 (32.8)	72 (28.5)	0.58
Secondary/technical	137 (56.1)	151 (59.7)	
University	27 (11.1)	30 (11.9)	

Abbreviation: SD, standard deviation.

^a^ Continuous variables: *t*‐tests for mean differences; and categorical variables: *χ* ^2^ test.

### 3.2 The impact of COVID‐19 on households

#### 3.2.1 Household livelihoods and coping mechanisms

**Figure 1:**
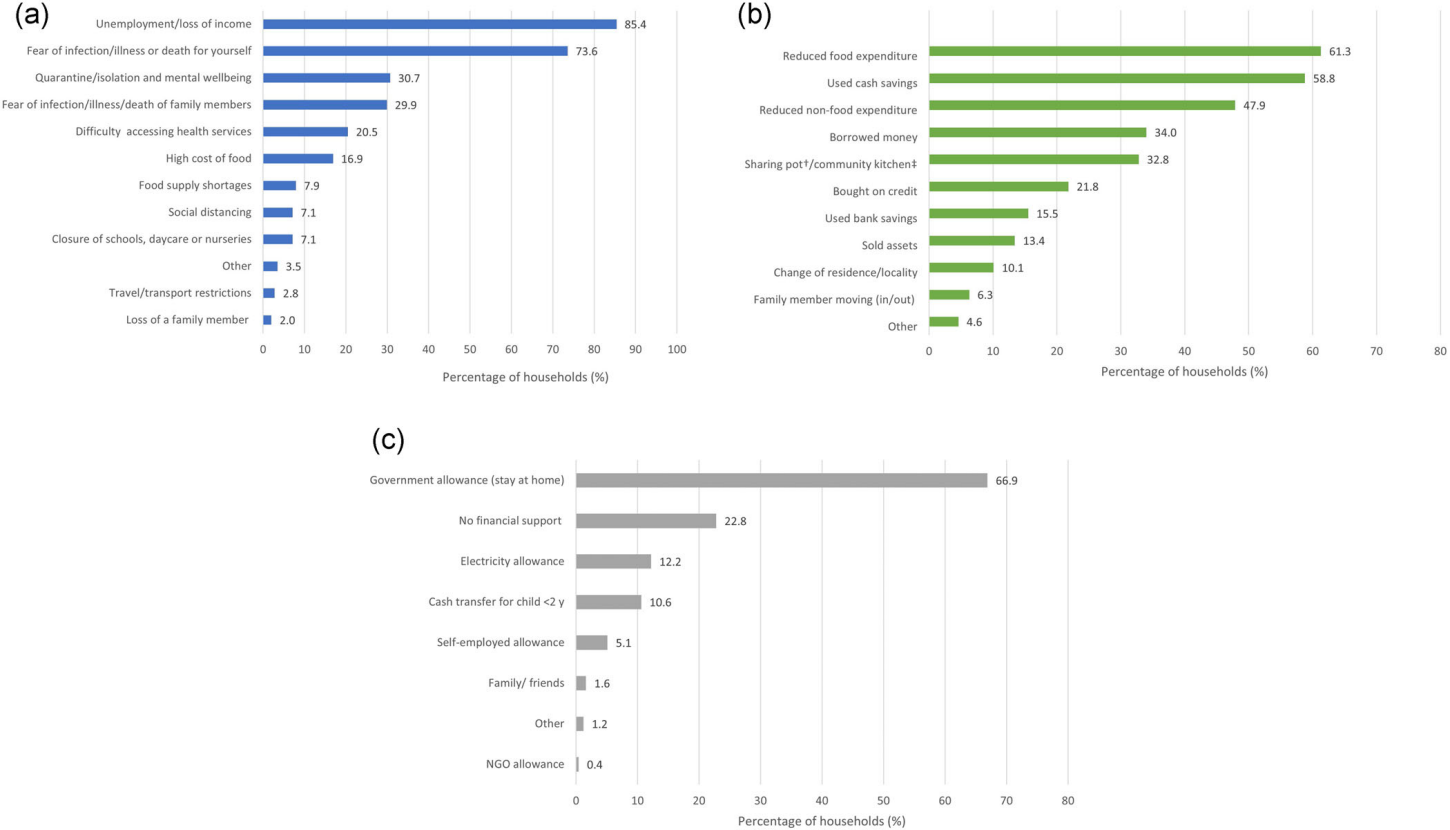
The impact of COVID‐19 on household livelihoods and strategies to deal with the financial impacts of the pandemic. (a) Concerns about the effects of the pandemic (*n* = 254) (top left figure). (b) Household strategies to manage the financial impacts of COVID‐19 (*n* = 238) (top right figure). †Shared cooking between neighbours. ‡Community spaces that receive food donations from the municipality or food programmes. (c) Sources of financial support received by households during the pandemic (*n* = 254) (bottom figure)

When asked to list up to three concerns about the effects of the pandemic, unemployment or loss of income was the most commonly reported concern (85.4%), followed by fear of infections/illness or mortality from coronavirus for the respondent (73.6%) and the impact of quarantine (stay at home orders) on mental wellbeing (30.7%) (Figure 1a). Concerns related to the difficulty of accessing health services, high cost of food, and food supply shortages were reported by 20.5%, 16.9%, and 7.9% of households, respectively.

Almost all respondents (98.0%) reported that the pandemic had an economic impact on their household, either a lot (72.0%) or to some extent (26.0%). Almost all respondents reported that their income was lower than before the pandemic (90.9%), with only three respondents (1.2%) reporting that income had increased since the pandemic. Almost all respondents (93.7%) said that they had made either a lot or some changes to household expenditure because of the economic impact of the pandemic.

Of the households that had made changes to adjust to the financial impact (*n* = 238), the most commonly reported strategy was to reduce food expenditure (61.3%). This was followed by using cash savings (58.8%); reducing nonfood expenditure (47.9%); borrowing money (34.0%), and making use of community kitchens or sharing pots (shared cooking between neighbours) (32.8%) (Figure 1b).

#### 3.2.2 Access to financial and food assistance programmes

The main form of financial support that was implemented in response to the pandemic was the government cash transfer associated with the stay‐at‐home order, received by 66.9% of households. About one in five households (22.8%) received no form of financial support and 10.6% received a cash transfer for a child under 2 years. Approximately, 12% of households received an electricity cash transfer. Very few households (less than 2%), reported receiving any financial support from friends, family, or nongovernmental organisations (NGOs) (Figure 1c).

Prepandemic, 36.5% of respondents reported receiving some form of food assistance through government social programmes. During the pandemic, this increased to 59.4% of respondents. The most common types of food assistance during the pandemic were municipality food baskets, received by 47.0% of households who received food support; the Qaliwarma programme (meals provided by schools to primary school children, which were changed to food baskets during the pandemic due to school closures) (25.2%); and the Vaso de Leche (municipal provision of food, usually oats, milk, and sometimes sugar) (15.9%). Other sources of food assistance included food baskets from the Cuna Más (government‐led preschool nurseries), donations from religious institutions, other government organisations, and NGOs. The full list of food support received pre‐ and during COVID‐19 is in Supporting Information Appendix 2.

#### 3.2.3 Access to and use of health services

Most caregivers reported that their access to health services had been affected, either a lot (73.6%) or slightly (18.9%). Only 5.5% thought that health services had not been affected, and five respondents stated that they did not know (2.0%). Most respondents (75.6%) reported that they had taken their child to a health facility in the past month for routine well‐child clinics for either growth and development checks (16.1%), immunisation appointments (33.1%), or both (26.4%). Of the respondents who reported they had not taken their child to a well‐child clinic appointment in the previous month (*n* = 62), the most common reasons were fear of infection/transmission of COVID‐19 (30.6%); the perception that the centres were only open for emergencies (29.0%); and fear of infection during transport/travel to the health centre (9.7%) (Supporting Information Appendix 3).

Approximately one‐fifth (20.9%) of the sample had received some form of remote consultation relating to health or nutrition consultation from the government health centre since the start of the pandemic. Of these remote consultations, most were by telephone (83.3%) or WhatsApp (15.2%). Of those who reported having a consultation by telephone/messaging, 60.6% reported receiving information on anaemia and iron supplementation, and 37.9% of caregivers reported receiving information on complementary feeding.

### 3.3 Impact of COVID‐19 on diet and food insecurity in mothers and IYC

#### 3.3.1 Household food insecurity during the pandemic

Using the FIES, 46.9% of households experienced moderate or severe household food insecurity, with 4.1% of households experiencing severe household food insecurity.

#### 3.3.2 Impact of COVID‐19 on IYC feeding practices

3.3.2.1

*Breastfeeding indicators (0–23 months)*: Overall, breastfeeding indicators were favourable in both the pre‐COVID‐19 and COVID‐19 samples, apart from exclusive breastfeeding under 6 months (Table 2). Almost all IYC were “ever breastfed” in both samples (>98.0%) and received colostrum (>98.0%). Early initiation of breastfeeding was relatively high in both samples (approximately 75% of mothers). Exclusive breastfeeding prevalence was much higher during the COVID‐19 pandemic compared to pre‐COVID‐19 (adjusted odds ratio, AOR = 3.79 [1.41–10.19], *p* = 0.008), despite remaining suboptimal (39.0%). However, the confidence intervals were wide. Most mothers (>75.0%) practised continued breastfeeding of IYC aged 12–23 months.

**Table 2:** Comparative table on nutrition indicators for mother–infant dyads before and during the COVID‐19 pandemic

			Crude analyses (STAMINA vs. PERUSANO)		Adjusted analyses^a^ (STAMINA vs. PERUSANO)	
	PERUSANO (*n* = 244) pre‐COVID‐19	STAMINA (*n* = 254) COVID‐19	*β* (SE)/OR [CI]	*p*	*β* (SE)/OR [CI]	*p*
Outcome variables	*n* (%)/mean (SD)	*n* (%)/mean (SD)				
**Infant and young child feeding indicators**						
*Breastfeeding indicators (0–23 months)*						
Child ever breastfed^b^	243 (99.6)	250 (98.8)	_	_	_	_
Child received colostrum^b^	241 (99.2)	247 (98.0)	_	_	_	_
Early initiation of breastfeeding	182 (74.6)	183 (72.6)	0.81 [0.33–1.99]	0.64	0.66 [0.25–1.73]	0.40
Exclusive breastfeeding (<6 months)	59 (24.2)	99 (39.0)	4.31 [1.68–11.08]	0.002	3.79 [1.41–10.19]	0.008
Continued breastfeeding 12–23 months	126 (78.3)	131 (76.6)	0.89 [0.51–1.57]	0.71	0.67 [0.24–1.89]	0.45
*Complementary feeding indicators (6–23 months)*						
Introduction of solid, semisolid, or soft foods (6–8 months)	35 (94.6)	39 (90.7)	0.54 [0.08–3.42]	0.51	0.85 [0.11–6.30]	0.87
Dietary diversity score	5.8 (1.2)	6.0 (1.2)	0.19 (0.11)	0.07	0.18 (0.11)	0.09
Minimum dietary diversity	208 (86.0)	227 (89.4)	1.41 [0.78–2.54]	0.25	1.38 [0.78–2.43]	0.27
Minimum meal frequency	210 (86.8)	215 (84.6)	0.87 [0.48–1.54]	0.63	0.87 [0.35–2.18]	0.78
Minimum milk feeding frequency for non‐breastfed children	25 (66.0)	22 (54.0)	0.60 [0.24–1.49]	0.27	0.69 [0.25–1.88]	0.47
Minimum acceptable diet	174 (71.9)	185 (72.8)	1.08 [0.69–1.71]	0.72	1.13 [0.70–1.85]	0.59
Egg and/or flesh food consumption	215 (88.8)	229 (90.2)	1.15 [0.65–2.04]	0.63	1.12 [0.61–2.05]	0.72
Zero vegetable or fruit consumption	9 (3.7)	4 (1.6)	0.41 [0.12–1.36]	0.15	0.48 [0.14–1.67]	0.25
Unhealthy food consumption	86 (35.5)	49 (19.3)	0.43 [0.29–0.65]	<0.001	0.40 [0.24–0.67]	<0.001
*Savoury and fried snack consumption* b	11 (4.5)	8 (3.1)	_	_	_	_
*Sweet foods*	80 (33.1)	46 (18.1)	0.45 [0.29–0.68]	<0.001	0.43 [0.26–0.71]	0.001
Sugar‐sweetened beverage consumption	196 (81.0)	200 (78.7)	0.87 [0.55–1.35]	0.53	0.72 [0.44–1.17]	0.19
**Maternal nutrition indicators**						
Dietary diversity score	5.7 (1.5)	5.5 (1.4)	−0.14 (0.13)	0.27	−0.08 (−0.13)	0.50
Minimum dietary diversity	188 (77.0)	191 (75.2)	0.89 [0.57–1.41]	0.64	0.92 [0.59–1.47]	0.75
Egg and/or flesh food consumption	232 (95.1)	239 (94.1)	0.10 [0.01–1.67]	0.11	0.70 [0.26–1.93]	0.49
Zero vegetable or fruit consumption	12 (4.9)	11 (4.3)	0.87 [0.38–2.02]	0.75	0.80 [0.34–1.89]	0.62
Unhealthy food consumption	96 (39.4)	62 (24.4)	0.43 [0.26–0.72]	0.001	0.48 [0.29–0.79]	0.004
*Savoury and fried snack consumption*	24 (9.8)	33 (13.0)	1.37 [0.77–2.41]	0.28	1.44 [0.80–2.59]	0.22
*Sweet foods*	83 (34.0)	37 (14.6)	0.32 [0.19–0.54]	<0.001	0.35 [0.21–0.59]	<0.001
Sugar‐sweetened beverage consumption	236 (96.7)	248 (97.6)	1.40 [0.48–4.09]	0.54	1.11 [0.35–3.56]	0.86

Abbreviations: CI, confidence interval; OR, odds ratio; SD, standard deviation; SE, standard error; *β*, *β* coefficient.

^a^ For infants, models were adjusted for child age, maternal education, household socioeconomic status, maternal occupation, and place of residence. For mothers, models were adjusted for maternal age, maternal education, household socioeconomic status, maternal occupation, and place of residence.

^b^ Odds ratios could not be obtained because of very low variability in the outcome variables.

3.3.2.2

*Complementary feeding indicators (6–23 months)*: Overall, complementary feeding indicators, including MDD, MMF, MAD, consumption of eggs and/or flesh foods, and zero consumption of fruits/vegetables were favourable (Table 2). The prevalence of timely introduction of solid, semisolid, and soft foods (6–8 months) was high in both samples (>90.0%) but lower during COVID‐19 compared to pre‐COVID‐19 (AOR = 0.85 [0.11–6.30], *p* = 0.87). The proportion of children who consumed a minimally diverse diet was high in both samples (>85.0%). The MDD score was 5.8 (1.2) in the pre‐COVID‐19 sample versus 6.0 (1.2) in the COVID‐19 sample, out of the eight predefined groups (adjusted *β* = 0.18 [0.11], *p* = 0.09). The proportion of children meeting the MMF was high in both samples (∼85.0%). The proportion of infants who achieved the MAD was similar in both samples at ∼72.0%. The consumption of eggs and/or flesh foods was high and similar across both samples (∼89.0%). A very small proportion of IYC did not consume any vegetables or fruit the day preceding the interview (3.7% pre‐COVID‐19% and 1.6% during COVID‐19). The consumption of unhealthy foods (i.e., savoury/fried snacks and/or sweet foods) was quite high (35.5% pre‐COVID‐19 vs. 19.3% during COVID‐19), with sweet foods contributing the most to this category. We observed a much lower prevalence of sweet food consumption during COVID‐19 (18.1%) in comparison to pre‐COVID‐19 (33.1%) (AOR = 0.43 [0.26–0.71], *p* = 0.001). The consumption of sugar‐sweetened beverages was very high (>78.0%) across both samples. Model fit statistics for infant dietary indicators are provided in Supporting Information Appendix 4.

3.3.2.3

**Figure 2:**
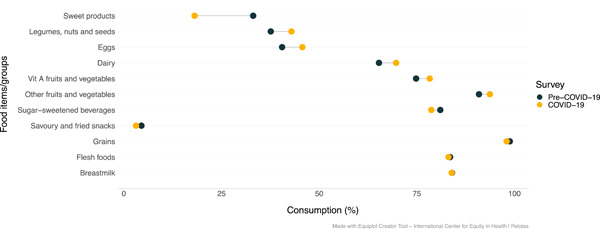
Child food consumption according to the survey (before vs. during COVID‐19) (*n* = 458)

*Food consumption*: Overall, the consumption of nutrient‐rich foods amongst IYC was higher during COVID‐19 in comparison to pre‐COVID‐19 (Figure 2). Indeed, the proportion of IYC who consumed legumes, nuts, and seeds; eggs; dairy products; vitamin A‐rich fruits and vegetables; and other fruits and vegetables was higher during COVID‐19. Food groups for which consumption was lower during the pandemic included sweet foods and savoury/fried snacks.

#### 3.3.3 Impact of COVID‐19 on maternal dietary outcomes

Maternal dietary indicators, including MDD, egg and/or flesh food consumption and zero vegetable or fruit consumption were favourable (Table 2). The consumption of unhealthy foods and beverages, however, was high in both samples. The proportion of women who met MDD was high (>75.0%) in both samples. The mean dietary diversity score was 5.7 (1.5) pre‐COVID‐19 versus 5.5 (1.4) during COVID‐19 (adjusted *β* = −0.08 [−0.13], *p* = 0.50). The consumption of eggs and/or flesh foods was very high (>94.0%) in both samples. A very small proportion of women in both samples did not consume any vegetables or fruit the day preceding the interview (∼4.5%).

The consumption of unhealthy foods was quite high, especially sweet foods. The proportion of mothers who consumed sweet foods was lower during COVID‐19 in comparison to pre‐COVID‐19 (14.6% vs. 34.0%) (AOR = 0.35 [0.21–0.59], *p* < 0.001). Almost all mothers consumed sugar‐sweetened beverages (>96.5%) and this was similar across both surveys. Model fit statistics for maternal dietary indicators are provided in Supporting Information Appendix 4.

**Figure 3:**
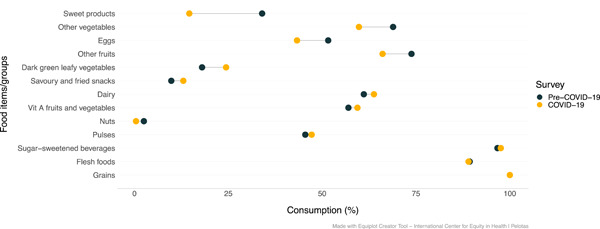
Maternal food consumption according to the survey (before vs. during COVID‐19) (*n* = 458)

The proportion of mothers who consumed dark green leafy vegetables, dairy products, other vitamins A‐rich fruit and vegetables, and savoury/fried snacks was higher during COVID‐19 in comparison to pre‐COVID‐19 (Figure 3). Food groups for which consumption was lower during the pandemic included sweet foods, other vegetables, eggs, other fruits, and nuts.

## 4 DISCUSSION

### 4.1 Summary and interpretation of findings

This study aimed primarily to assess whether the pandemic influenced diets of mothers and IYC in two low‐income urban areas of Peru, as well as capture wider contextual factors surrounding maternal and IYC diets during COVID‐19. Almost half of the households in the survey (46.9%) were at risk of moderate or severe household food insecurity. The most recent comparable national data available pre‐dating the pandemic estimated a prevalence of 29.9% moderate or severe household food insecurity in 2015 using the same survey tool (FIES) (World Bank, 2021). A national survey of urban and rural samples in January 2021 reported a food insecurity prevalence of 42.9% moderate and 12.0% severe, using a different assessment tool (consolidated approach to reporting on FS indicators) (World Food Programme, 2021), hence prevalence rates may not be directly comparable. Although we have no pre‐COVID assessment of food insecurity in the same study sites, concerns about unemployment and loss of income due to COVID‐19 reported by a high proportion of respondents very likely contributed to household food insecurity. National data showed a 9.9% increase in the population living in monetary poverty from 20.2% to 30.1% from 2019 to 2020 (INEI, 2020b). A national survey in Mexico found that food insecurity levels increased by 25% between June 2018 and 2020 (Gaitán‐Rossi et al., 2021). Government responses in Peru to the pandemic included financial and food assistance programmes to vulnerable populations which may have ameliorated economic impacts. In our sample, the proportion of households receiving food assistance increased from 36.5% prepandemic to 59.4% during the pandemic. More research is warranted on the role of informal food support systems, such as the shared pot or community kitchens used by nearly one‐third (32.8%) of respondents, and how these contribute to diet and/or prevention of food insecurity.

Despite these economic and financial shocks, we found few changes in dietary indicators of mothers and their IYC. In contrast, other studies have reported nutritional impacts of COVID‐19 such as a shift from more nutritious food groups to cheaper foods, often staples, for example, in Kenya and Uganda (Kansiime et al., 2021) and Ethiopia (Hirvonen et al., 2021) but data are still limited. Our findings in Peru may suggest that IYC and maternal diets are resilient to the social and economic effects of the pandemic or were buffered by the financial or food assistance received (by 77.0% and 56.0% of households, respectively). In other LMICs, there is some evidence that social programmes have provided an important buffer for nutritional indicators, but such programmes vary greatly by context and country (Picchioni et al., 2021). Alternatively, the dietary indicators in our study may not be sufficiently sensitive to detect worsening diet quality. Although we found that 80.0% of IYC met the MDD, this does not reflect quantitative dietary intakes, which is important in contexts such as Peru where dietary diversity is typically high, but intake of critical nutrients may be inadequate, as illustrated by the high prevalence of anaemia (INEI, 2021). Similarly, although ∼85% of IYC met MMF suggesting that most were meeting energy requirements, this does not guarantee that all nutrient requirements were met.

A positive change in infant nutrition from pre‐ to mid‐pandemic was the increase in exclusive breastfeeding from 24.2% to 39.0% (*p* < 0.008), although the strength of the association was weak. While we do not have any direct evidence, this change could be associated with the lower proportion of women who were working during the pandemic (21.7% during COVID‐19 compared to 32.8% prepandemic), leading to more time at home and potentially more time for breastfeeding. National data also show a slight upward trend for exclusive breastfeeding reported at 68.4% in 2020 compared to 65.6% in 2019 (INEI, 2021). A second positive change was the marked decrease in the proportion of IYC consuming unhealthy foods (i.e., savoury/fried snacks and sweet foods) from 35.5% to 19.3% (*p* < 0.001) and among mothers, for sweet foods only (34.0% to 14.6%, *p* < 0.001). Around 61.0% of households reported reducing household food expenditure due to the pandemic which may have led to reduced purchasing of unhealthy foods. Alternatively, periods of lockdown or restrictions may have led to reduced exposure to unhealthy food environments or social activities associated with the consumption of unhealthy foods. The high prevalence of sugar‐sweetened beverage consumption, consumed by ∼78% of IYC and ∼97% of mothers, was unchanged between the two surveys and is a concern for dietary health. Sweetened beverages in these communities are commonly home‐prepared drinks rather than commercially produced beverages (Kovalskys et al., 2019) which may explain the consistently high prevalence.

### 4.2 Strengths and limitations

Strengths of this study include nutritional assessments conducted in the same two low‐income communities in the months immediately before as well as during the pandemic, using the same interview teams and assessment instruments. While data collection methods differed (face‐to‐face interviews in the first survey vs. telephone interviews in the second), we used the same interviewers, all of whom had close knowledge of the communities studied. Telephone interviews could potentially have excluded households without mobile phone access, but since 95% of households nationally have access to a mobile phone (INEI, 2020b) this is unlikely to be a major source of selection bias. Limitations of the study include the use of population‐level nutrition indicators, such as MDD, MMF, and MAD, which provide a broad assessment of dietary quality but are not designed to detect quantitative changes in food intake. A quantitative dietary assessment, capturing data on portion sizes or weighed food intakes, would be required to identify any changes in quantities of food consumed or nutrient adequacy. This type of dietary assessment would be challenging to conduct via telephone interviews because of the length of interviews and the need to assess portion sizes via visual prompts. We had no a priori sample size calculation because the impact of COVID‐19 on dietary indicators was unknown at the onset of the pandemic when we developed the STAMINA study. However, we hypothesised that a reduction of 10% points in the MDD prevalence from PERUSANO to the STAMINA survey would indicate a notable detriment to the diets of mothers and infants. Using a post hoc power calculation with power at 80%, *α *at 5%, and the sample size in each survey (*n* = 244 and *n* = 254) with the pre‐COVID prevalence of meeting MDD, then we had the power to detect an 11% point reduction in maternal MDD (i.e., 77%– 66%) and a 10% point reduction in infant MDD (89%–79%). For some indicators, such as the FIES, we did not collect data before the pandemic and therefore cannot directly compare this indicator pre‐and during COVID‐19 in these communities. However, knowledge of household food insecurity during the pandemic is an important benchmark for future comparisons of the impact of COVID‐19 with changing economic and social conditions. This survey may not reflect the longer‐term consequences of the pandemic on the diets of mothers and IYC since the date of the survey.

## 5 CONCLUSION

Compared to prepandemic data, maternal and IYC dietary indicators had not significantly worsened 9 months into the COVID‐19 pandemic. Despite this, almost half of households were at risk of moderate‐severe household food insecurity. A high proportion of households received food assistance or government cash transfers during the pandemic which may have contributed to the stability of dietary indicators.

We identified maternal and IYC feeding indicators requiring improvement, such as reducing the consumption of sugar‐sweetened beverages, and the prevalence of unhealthy food consumption. These unhealthy dietary indicators could be targets for future policy.

The negative economic impact of the pandemic, and consequent reductions in household food expenditure, demonstrate the importance of providing foods of high dietary quality (i.e., nutrient‐rich) and variety through social programmes as a future policy recommendation. Informal food support systems, such as the shared pot, may also contribute to resilient dietary outcomes and warrant further exploration.

Implementation of double‐duty actions (i.e., actions that target multiple forms of malnutrition simultaneously) to improve the suboptimal nutritional indicators and address multiple forms of malnutrition in the population will be particularly important as populations recover from the COVID‐19 pandemic.

## CONFLICT OF INTERESTS

The authors declare no conflict of interest.

## AUTHOR CONTRIBUTIONS

EKR, RP, HMC‐K, RP, PLG, and NV designed the research studies. EKR, RP, HMC‐K, RP, PLG, NV, MH, and EL were involved in designing the data collection approach and tools. RP and HMC‐K led data collection and performed data quality checks. RP, RP, EKR, SE‐D, and MS analysed the data. RP and EKR wrote the first draft of the paper with critical input from Rossina Pareja. All authors reviewed the manuscript and approved the final version.

## Supporting information

Supporting information.

## Data Availability

The survey questionnaires and data that support the findings are available as open access resources via the Loughborough University Research Repository for the PERUSANO study (at 10.17028/rd.lboro.18750458 and 10.17028/rd.lboro.16691455) and the STAMINA study (10.17028/rd.lboro.16825507 and 10.17028/rd.lboro.18785666) following a period of embargo.
