# Supplementary material for: Diet and food insecurity among mothers, infants, and young children in Peru before and during COVID‐19: A panel survey
Source: Matern Child Nutr. 2022 Mar 11;18(3):e13343. doi: 10.1111/mcn.13343 (PMC9115223; doi:10.1111/mcn.13343)
Supplement: Supplementary file 1 — Supporting information. [file MCN-18-e13343-s001.docx]

**Supporting information**

Supplementary appendix 1: Dietary indicator definitions^†^

| **Infant and young child indicators** | |
| --- | --- |
| **Indicator (abbreviation)** | **Definition** |
| Ever breastfed (EvBF) | Percentage of children who were ever breastfed |
| Early initiation of breastfeeding (EIBF) | Percentage of children who were put to the breast within one hour of birth |
| Exclusive breastfeeding under 6 months (EBF) | Percentage of infants who were fed exclusively with breast milk from 0–5 months of age (*via retrospective recall*) |
| Continued breastfeeding 12-23 months (CBF) | Percentage of children who were fed breast milk from 12–23 months of age (*via retrospective recall*) |
| Introduction of solid, semi-solid or soft foods | Percentage of infants who consumed solid, semi-solid or soft foods from 6–8 months of age (*via retrospective recall*) |
| Dietary diversity score (DDS) 6-23 months | Mean number of food groups consumed out of 8 pre-defined food groups during the previous day |
| Minimum dietary diversity (MDD) 6-23 months | Percentage of children 6–23 months of age who consumed foods and beverages from at least five out of eight defined food groups during the previous day |
| Minimum meal frequency (MMF) 6-23 months | Percentage of children 6–23 months of age who consumed solid, semi-solid or soft foods (but also including milk feeds for non-breastfed children) the minimum number of times  or more during the previous day |
| Minimum acceptable diet (MAD) 6-23 months | Percentage of children 6–23 months of age who consumed a minimum acceptable diet during the previous day |
| Egg and/or flesh food consumption (EFF) 6-23 months | Percentage of children 6–23 months of age who consumed egg and/or flesh food during the previous day |
| Sweet beverage consumption (SwB) 6-23 months | Percentage of children 6–23 months of age who consumed a sweet beverage during the previous day |
| Unhealthy food consumption (UFC) 6-23 months | Percentage of children 6–23 months of age who consumed selected sentinel unhealthy foods* during the previous day |
| Zero fruit and vegetable consumption (ZVF) 6-23 months | Percentage of children 6–23 months of age who did not consume any vegetables or fruits during the previous day |
| **Maternal indicators** | |
| **Indicator (abbreviation)** | **Definition** |
| Maternal dietary diversity score  (MDD-S) | Mean number of food groups consumed out of 10 pre-defined food groups during the previous day |
| Minimum adequate dietary diversity (MDD-W) | Consumption of at least five of 10 pre-defined food groups |

‡ Adapted from: (FAO and FHI 360, 2016; WHO and UNICEF, 2021)

* Selected sentinel unhealthy foods (WHO and UNICEF, 2021) defined as: Candies, chocolate and other sugar confections, including those made with real fruit or vegetables like candied fruit or fruit roll-ups; frozen treats like ice cream, gelato, sherbet, sorbet, popsicles or similar confections; cakes, pastries, sweet biscuits and other baked or fried confections which have at least a partial base of a refined grain, including those made with real fruit or vegetables or nuts, like apple cake or cherry pie; chips, crisps, cheese puffs, French fries, fried dough, instant noodles and similar items which contain mainly fat and carbohydrate and have at least a partial base of a refined grain or tuber. These foods are also often high in sodium (WHO and UNICEF 2021).

**References**

FAO and FHI 360. (2016). *Minimum Dietary Diversity for Women: A Guide For Measurement*. *Minimum Dietary Diversity for Women: A Guide for Measurement*. Rome. https://doi.org/10.1016/S0167-6393(00)00055-8

WHO and UNICEF. (2021). *Indicators for assessing infant and young child feeding practices: definitions and measurement methods*. Geneva.

**Supplementary appendix 2.** Programmes or institutions from which respondents received food assistance ^1,2^

|  | PERUSANO  (n=89) | STAMINA  (n=151) | | |
| --- | --- | --- | --- | --- |
|  | n (%) | n (%) |  |  |
| Municipality food basket | 0 (0) | 71 (47.0) | | |
| Qaliwarma† | 29 (32.6) | 38 (25.2) | | |
| Vaso de leche ‡ | 75 (84.3) | 24 (15.9) | | |
| Cuna Más food basket* | 5 (5.6) | 8 (5.3) | | |
| Friends/ family | 0 (0) | 7 (4.6) | | |
| NGO | 0 (0) | 6 (4.0) | | |
| Community kitchens (Comedor popular) | 4 (4.5) | 2 (1.3) | | |
| Government institutions | 0 (0) | 19 (12.6) | | |
| Religious organisations | 0 (0) | 6 (4.0) | | |
| Other | 0 (0) | 3 (2.0) | | |

^1^ Multiple responses were given hence percentages add up to more than 100% and number of responses is greater than total number of respondents

^2^ PERUSANO includes 89 respondents who reported receiving some form of food assistance out of total sample of 244 respondents. STAMINA includes 151 out of 254 respondents who reported that they had received some form of food assistance since the pandemic

† Qaliwarma programme provides meals to school children. During COVID-19 these were delivered to homes because of school closure during the pandemic.

‡ Vaso de leche is the municipal provision of food, usually oats, milks and sometimes sugar.

* Cuna Más food basket is a food assistance programme delivered through government-led pre-school nurseries. During COVID-19 these were delivered to households while nurseries were closed.

**Supplementary appendix 3.** Reasons for not taking infant or young child to well-baby appointments (CRED) in the last month (n=62)^1^

|  | n (%) |
| --- | --- |
| Reluctance to go to the health facility because of fear of contamination | 19 (30.6) |
| Health facility closed/only open for emergencies | 18 (29.0) |
| Fear of contamination whilst commuting to the health facility | 6 (9.7) |
| Lack of time | 5 (8.1) |
| No appointment due | 5 (8.1) |
| Relatives/friends advised not to go | 4 (6.5) |
| Centre is providing telephone consultation only | 3 (4.8) |
| Had a home visit | 2 (3.2) |
| Lack of transport | 1 (1.6) |
| Mother or other member in the family ill with COVID-19 (isolation, quarantine) | 1 (1.6) |
| Other | 7 (11.3) |

^1^ Includes 62 out of 254 respondents who reported that they had not taken their child to the health centre for an appointment

**Supplementary appendix 4**

|  | Model fit statistics | | |
| --- | --- | --- | --- |
|  | LL | AIC | BIC |
| **Infant and young child feeding indicators** |  |  |  |
| ***Breastfeeding (0-23 months)*** |  |  |  |
| Early initiation of breastfeeding | -255.48 | 526.96 | 560.59 |
| Exclusive breastfeeding (<6 months) | -274.38 | 564.76 | 598.43 |
| Continued breastfeeding 12-23 months | -159.44 | 334.88 | 365.29 |
| ***Complementary feeding (6-23 months)*** |  |  |  |
| Introduction of solid, semi-solid or soft foods  (6-8 months) | -18.37 | 52.76 | 71.81 |
| Dietary diversity score | -781.19 | 1580.39 | 1618.23 |
| Minimum dietary diversity | -173.37 | 362.75 | 396.38 |
| Minimum meal frequency | -179.89 | 375.79 | 409.43 |
| Minimum milk feeding frequency for non-breastfed children | -47.95 | 111.89 | 130.85 |
| Minimum acceptable diet | -281.63 | 579.25 | 612.89 |
| Egg and/or flesh food consumption | -157.45 | 330.89 | 364.53 |
| Zero vegetable or fruit consumption | -52.81 | 121.62 | 155.26 |
| Unhealthy food consumption | -256.67 | 529.35 | 562.35 |
| Sugar-sweetened beverage consumption | -211.61 | 439.23 | 472.87 |
| **Maternal nutrition indicators** |  |  |  |
| Dietary diversity score | -879.75 | 1777.50 | 1815.38 |
| Minimum dietary diversity | -266.83 | 549.66 | 583.33 |
| Egg and/or flesh food consumption | -99.84 | 215.68 | 249.35 |
| Zero vegetable or fruit consumption | -91.50 | 199.00 | 232.67 |
| Unhealthy food consumption | -297.85 | 611.70 | 645.37 |
| Sugar-sweetened beverage consumption | -56.37 | 128.75 | 162.42 |

LL: log-likelihood; AIC: Akaike information criterion; BIC: Bayesian information criterion
